# Evaluating exposure and potential effects on honeybee brood (*Apis mellifera*) development using glyphosate as an example

**DOI:** 10.1002/ieam.1529

**Published:** 2014-02-25

**Authors:** Helen M Thompson, Steven L Levine, Janine Doering, Steve Norman, Philip Manson, Peter Sutton, Georg von Mérey

**Affiliations:** † FERASand Hutton, York, United Kingdom; ‡ Monsanto CompanySt. Louis, Missouri, USA; § Feinchemie Schwebda GmbHCologne, Germany; || Dow AgroSciencesAbingdon, Oxfordshire, United Kingdom; # Cheminova A/SCardale Park, Harrogate, United Kingdom; †† Syngenta, Jealott's Hill International Research CentreBracknell, Berkshire, United Kingdom; ‡‡ VerbarlMonsanto Europe SABrussels, Belgium; §§ Present address is Syngenta, Jealott's Hill International Research CentreBracknell, Berkshire, United Kingdom; ## Present address is RidgewayEco, Innovation CentreMilton Park, Abingdon, Oxfordshire, United Kingdom

**Keywords:** Brood, Glyphosate, Honeybee, Toxicity testing, Pesticides

## Abstract

This study aimed to develop an approach to evaluate potential effects of plant protection products on honeybee brood with colonies at realistic worst-case exposure rates. The approach comprised 2 stages. In the first stage, honeybee colonies were exposed to a commercial formulation of glyphosate applied to flowering *Phacelia tanacetifolia* with glyphosate residues quantified in relevant matrices (pollen and nectar) collected by foraging bees on days 1, 2, 3, 4, and 7 postapplication and glyphosate levels in larvae were measured on days 4 and 7. Glyphosate levels in pollen were approximately 10 times higher than in nectar and glyphosate demonstrated rapid decline in both matrices. Residue data along with foraging rates and food requirements of the colony were then used to set dose rates in the effects study. In the second stage, the toxicity of technical glyphosate to developing honeybee larvae and pupae, and residues in larvae, were then determined by feeding treated sucrose directly to honeybee colonies at dose rates that reflect worst-case exposure scenarios. There were no significant effects from glyphosate observed in brood survival, development, and mean pupal weight. Additionally, there were no biologically significant levels of adult mortality observed in any glyphosate treatment group. Significant effects were observed only in the fenoxycarb toxic reference group and included increased brood mortality and a decline in the numbers of bees and brood. Mean glyphosate residues in larvae were comparable at 4 days after spray application in the exposure study and also following dosing at a level calculated from the mean measured levels in pollen and nectar, showing the applicability and robustness of the approach for dose setting with honeybee brood studies. This study has developed a versatile and predictive approach for use in higher tier honeybee toxicity studies. It can be used to realistically quantify exposure of colonies to pesticides to allow the appropriate dose rates to be determined, based on realistic worst-case residues in pollen and nectar and estimated intake by the colony, as shown by the residue analysis. Previous studies have used the standard methodology developed primarily to identify pesticides with insect-growth disrupting properties of pesticide formulations, which are less reliant on identifying realistic exposure scenarios. However, this adaptation of the method can be used to determine dose–response effects of colony level exposure to pesticides with a wide range of properties. This approach would limit the number of replicated tunnel or field-scale studies that need to be undertaken to assess effects on honeybee brood and may be of particular benefit where residues in pollen and nectar are crop- and/or formulation-specific, such as systemic seed treatments and granular applications. *Integr Environ Assess Manag* 2014;10:463–470.

## INTRODUCTION

Studies on the effects of pesticides on honeybee colonies now require consideration of the effects on developing brood as a standard requirement (USEPA 2012; EFSA 2013). Several approaches are recommended (EFSA 2013) but each has its own associated problems. Currently, the most optimal approach is that described in the Organisation for Economic Co-operation and Development (OECD) 75 guidance document that assesses the effects on brood in colonies confined in tunnels to the treated crop. Although replication can be readily achieved, the costs of running multiple exposure scenarios, e.g., across crops or treatment rates, is prohibitive and such studies are also technically challenging due to the limited forage available and adverse effects of confinement. The in vitro larval method (Aupinel et al. 2007) is applicable for routine larval toxicity screening but currently cannot be used to reliably evaluate effects on emergence due to high background pupal mortality. Furthermore, this laboratory method does not take into account effects on brood care by worker bees and it is unclear how to link residues in nectar or pollen with concentrations tested in brood food. The alternative colony-scale feeding study (Oomen et al. 1992) has been limited in its approach by the use of exposure levels only related to application rate, as it was originally devised solely to investigate adverse effects of insect growth-regulating pesticides. The Oomen et al. (1992) study may be improved by linking the dosing rates used in the feed to those encountered by foraging bees, so that a dose–response design can be developed to allow interpretation of effects at multiple dose levels in a single study. Results from a single study may thus be applied to a range of differing application rates and/or crops based on residue data. The aim of this study was to develop the Oomen et al. (1992) approach using a model pesticide, glyphosate, for demonstration of exposure. The first stage quantified residues in relevant matrices (pollen, nectar, and larvae), when a glyphosate-based formulation was applied to flowering *Phacelia* in large glasshouses in which honeybee colonies were confined. Pollen and nectar residue data were then used to predict the total exposure (i.e., dose rate) of the colony to glyphosate based on foraging rates and food requirements of the colony. This dose rate was used in a brood feeding test by feeding treated sucrose solution directly to honeybee colonies, and the effects of exposure and subsequent residues in larvae were compared.

## MATERIALS AND METHODS

Technical grade glyphosate (62.27% w/w glyphosate isopropylamine [IPA] salt corresponding to 46.14% w/w glyphosate acid equivalent [a.e.]) and the soluble concentrate formulation of glyphosate (MON 52276) (30.68% glyphosate a.e. as the IPA salt, batch no GLP-0810-19515-A), supplied by Monsanto (St. Louis, MO) were used in the study. All honeybee colonies were obtained from National Bee Unit, FERA, (York, UK) apiaries and were confirmed as having low incidence of adult bee diseases, viruses, and varroa with no clinical signs of brood diseases.

### Exposure assessment

Two 180 m^2^ well-ventilated but insect-proof glasshouses were used for the study so as to be as representative as possible of the outdoor situation (e.g., polytunnel) but without direct rainfall. *Phacelia* was planted directly into the soil in the glasshouses and no pesticides were used during its cultivation. Application was performed when *Phacelia* flowers were at 100% of full bloom.

Three days before the application, 2 small honeybee colonies comprised of 4 to 6 frames of brood and 6000 to 12 000 adult bees were located on opposite sides of each glasshouse and allowed to fly freely. At the time of installation, each colony was fitted with a pollen trap and provided with a limited amount of stores to ensure that feeding on the crop was encouraged. This was done by removing as many frames as possible which contain only nectar or pollen, while ensuring survival and a maximum foraging activity. A supply of clean water, with provision to prevent bees from drowning, i.e., a sponge, was provided and replenished as required (it was removed during spray application).

To confirm that bees were foraging on the flowering *Phacelia*, foraging assessments were carried out each day during times when peak activity was expected. The assessments were performed by marking a 5 m × 1 m wide transect within the crop and counting the number of bees foraging within the marked area during a 1 min period once each day during the peak activity period (between 10.00–15.00 h in this study, based on previous experience). In addition, the number of bees returning to each hive and the number carrying pollen loads were counted during a 30 s period. These 2 counts provided information on the level of foraging activity of each hive within each glasshouse. Daily assessments of the crop were undertaken by visual assessment of the quality of the forage available, e.g., % plants with wilted flowers, wilted leaves.

The glyphosate formulation was applied at a rate equivalent to 8 L/ha (2.88 kg a.e./ha) in 400 L water/ha achieving an application efficiency of between 102% to 104% of the target rate, in both glasshouses. The application rate of 2.88 kg a.e./ha is the highest single application rate recommended for glyphosate, whereas the typical single application rate is 2.16 kg a.e./ha. The final treatment solution was prepared by adding the required quantities of test item—measured by weight, to measured volumes of tapwater and thoroughly mixing in the field immediately before use to give the final treatment solution. The application was made during a period when the bees were actively foraging, using a 3 nozzle lunch box sprayer unit with a hand-held boom fitted with Lurmark 03 F110 nozzles. Direct spray drift onto the colonies was avoided by directing the spray away from the hives, and no direct overspray of the colonies occurred.

Pollen traps were activated 24 h before pollen collection, and the content of the pollen trap fitted to each hive was collected on days −1 (i.e., the day before application), 1, 2, 3, 4, and 7 after the application. The content of the traps was discarded on day 6 so as to only collect a sample from days 6 to 7. Each day and hive sample was kept separate unless they were too small for residue analysis, in which case samples from the same glasshouse were combined. All samples of pollen, nectar, and larvae were stored at −20° C.

On days 0 (before application), 1, 2, 3, 4, and 7 after the application samples of approximately 40 returning forager bees were collected from each colony by blocking the entrance of the hives with a foam bung and collecting returning foraging bees directly into collection jars. The nectar was collected from the honey stomachs of individual honeybees by removal of the stomach by dissection and placed in a preweighed tube. Samples were combined to produce samples large enough for residue analysis (minimum 200 mg).

On days 4 and 7 after the application, samples of 10 4–5-day-old larvae were taken from each colony using a forceps and stored at −20° C. Each day and hive sample was kept separate. On day 7, an additional sample of nectar was taken from the combs using a syringe in each colony and each hive sample was kept separate.

### Residue analysis

Residues of glyphosate were extracted from larvae, pollen, nectar, and sucrose solution samples with acetonitrile/water (1:4, v/v). Recovery samples were fortified by spiking blank samples after weighing. For larvae, pollen, and nectar, the whole sample was accurately weighed into a single-use centrifugation tube. The sample was then homogenized, extracted with acetonitrile–water (1:4) with a high speed laboratory mixer, separated by centrifugation followed by solid-phase extraction of the supernate using a C18 column. All samples were then derivatized with fluorenylmethyl-chloroformate (FMOC-Cl). For derivatization, internal standard (1.0 μg/mL), borate buffer (0.2 mol/L sodium tetraborate decahydrate in water), and FMOC-Cl (5 g/L in acetonitrile) were added to the diluted extract. The samples were closed, mixed, and incubated at ambient temperature for at least 1 h. Finally, pH 3 water was added.

A second cleanup was carried out by applying the derivatized product to an Oasis HLB SPE column (equilibrated with dichloromethane followed by methanol and pH 3 water) and then rinsed with dichloromethane and the glyphosate-FMOC was eluted with methanol. The eluate was evaporated to dryness using a vacuum rotary evaporator. The residue was reconstituted in 5% acetonitrile solution and transferred into a glass vial for high-performance liquid chromatography (HPLC)-tandem mass spectrometry (MS/MS) analysis.

The samples were analyzed using high-pressure liquid chromatography (Shimadzu LC-System) coupled with a triple quadrupole mass spectrometry detector (Sciex API4000). A Phenomenex Synergi column 2.5 μm Max-RP, 20 × 2.0 mm, 2.5 μm (No. 00M-4372-B0-CE) + 4 mm guard column was used. The column temperature was 40° C and a 30 μL injection volume was used. The mobile phase comprised A: water + 0.1% acetic acid (80%), B: methanol + 0.1% acetic acid (15%), and C: 100 mM ammonium acetate solution in methanol (5%) with a linear gradient over 5 min to comprise A: water + 0.1% acetic acid (0%); B: methanol + 0.1% acetic acid (95%) and C: 100 mM ammonium acetate solution in methanol (5%). Glyphosate-FMOC was quantified using the transition 390.0 to 149.8 with an internal standard glyphosate 1,2-^13^C2 15N-FMOC transition 393.0 to 152.8.

At the start of the analytical sequence, the detector linearity was confirmed over the calibration range of interest by constructing a calibration function of peak area versus concentration within the range from 2.0 ng/mL to 5000 ng/mL for larvae and nectar samples, 1.0 ng/mL to 3500 ng/mL for pollen samples, and from 2.0 ng/mL to 4000 ng/mL for sucrose solution samples. Injections of sample extracts were interspersed with injections of quality control standards after 2 to 4 samples to verify the detector response.

The methods were validated before use and showed 92%–102% recovery with relative standard deviation (RSD) <15% with sucrose samples spiked at 1 and 400 mg a.e./kg, larval samples spiked at 1 and 200 mg a.e./kg, pollen samples spiked at 1, 500 and 700 mg a.e./kg and nectar samples spiked at 1 and 500 mg a.e./kg. Calibrations were linear within the range. Unless otherwise specified the limit of detection (LOD) was 0.3 mg a.e./kg, denoted as not detected (n.d.), and the limit of quantitation (LOQ) was 1.0 mg a.e./kg. Where data were used to generate mean values residues less than the LOQ were ascribed a value of 0.6 mg a.e./kg.

### Effects assessment

Two approaches were made to assess exposure levels to be used in the effects study: one based on generic published data on the requirements for nectar and pollen by larvae (generic data) and the other based on the observations made in the exposure study (study data).

#### Generic data

The calculations were based on a daily brood requirement of 30 mg nectar (based on 40% sugar in nectar) and 1 mg pollen per worker larva (Rortais et al. 2005). Based on a brood frame being 3600 cells (British Standard frame) and 5 frames of brood (4–6 were used in this study), there are 18 000 brood cells. The brood is unsealed for 25% of the time (hatch day 3 to sealed day 8 with emergence day 21, empirically determined in this study) therefore 4500 larvae have a requirement for 135 g/d nectar and 4.5 g/d pollen.

#### Study data

The second approach was to assess the amount of pollen and nectar returning to the hive over the time course of exposure using the data on the numbers of returning foragers in the study and the amounts of pollen and nectar collected from bees by using the pollen trap and individual bee samples.

The maximum pollen collected per colony was 2.9 g on day 1 and the traps were estimated to be approximately 50% efficient based on calculated pollen collection (Levin and Loper 1984; Delaplane et al. 2013). Thus 6 g of pollen per day was returned to the hive (the colony was using approximately 4.5 g of this based on the study by Rortais et al. [2005]).

The nectar collection was more difficult to directly assess but with a mean of 18 foragers returning to the hive per 30 s (observed in this study) and approximately 50 μL per load (max) this gives 18 trips/30 s × 60 s/min × 60 min/h × 12 h max foraging/d = 25 920 trips/d × 0.050 mL = 1296 mL/day (of which the colony was using 135 g, based on Rortais et al. [2005]). Because the assessment is brood exposure, the conservative collection estimate is justified. Therefore, as a worst case example considering the colony size used in the exposure study, the colony collected 6 g pollen and 1296 mL (i.e., 518 g sugar, assuming 40% sugar content) nectar and of this the brood consumes 4.5 g pollen and 135 g nectar (Rortais et al. 2005) that allowed the excess to be stored for later consumption.

Considering that bee colonies used in the brood study were up to 50% bigger than those used in the residue study, an additional calculation for the expected total daily intake of glyphosate residues was undertaken assuming that such colonies would collect 9 g pollen and 1944 mL nectar (Table2). Furthermore, the determined residue content based on a worst-case application rate of 2.88 kg a.e./ha for spot treatments in orchards and vines and was adjusted to reflect the more realistic maximum application rate of 2.16 kg a.e./ha for preplanting, preemergence of crops, and preharvest applications.

The brood feeding study was undertaken using glyphosate as the technical grade IPA salt. Three dose levels of the test item were used based on the residues identified in pollen and nectar in a glass house study performed before the initiation of the bee brood study (Table1). The lowest dose was based on the mean residue concentrations achieved over the first 3 days following the residue study spray application (75 mg glyphosate a.e./L). The mid-dose was based on the highest residue concentrations following the spray application (150 mg glyphosate a.e./L) and the highest dose was equivalent to twice this latter rate (301 mg glyphosate a.e./L). The test item was introduced into each hive in equivalent volumes of 50% sucrose (w/v) solution (1 L) for each treatment group. Hence, the range could also be expressed in terms of concentration in the introduced dosing solution (mg glyphosate a.e./L and mg glyphosate a.e./kg). Control colonies were supplied with 50% w/v sucrose solution in deionized water and the toxic reference, fenoxycarb, (750 mg a.s./L as the formulation Insegar WG 250 g a.s./kg, batch no SM01A406) reported to have significant adverse effects on honeybee brood, was used to ensure that the study had the ability to detect effects of the test substance if they occurred (de Ruijter and van der Steen 1987).

**Table 1:** Summary of residue analyses of nectar collected from hive combs and larvae during the exposure study

Matrix	Hive	[mg glyphosate acid equivalent/kg]	
		4 days after treatment	7 days after treatment
Nectar directly from hive	A	—	<LOQ (<0.6)
	B	—	1.30
	C	—	1.06
	D	—	1.00
	Mean ± SE	—	0.99 ± 0.15
Larvae from hive^a^	A	8.32	2.54
	B	16.70	10.6
	C	19.50	6.72
	D	2.88	1.23
	Mean ± SE	11.9 ± 3.8	5.3 ± 2.1

a LOQ = 0.3 mg a.e./kg for 4-day-old larvae and LOQ = 1.0 mg a.e./kg for 7-day-old larvae.

Twenty standardized honeybee colonies each consisting of a single wooden Smith hive with British Standard frames and a queen were used; each of the queens used in the study was of similar age and lineage. The colonies were divided into 5 groups of 4 colonies. Each colony had a dead bee trap fitted to the front and the contents were counted daily during the brood assessment period (Imdorf et al. 1987). The colonies contained a mean of 14 250 to 19 500 adult bees, 1.5 to 2.5 frames of brood, 1.0 to 1.9 frames of stores, and 0.2 to 0.7 frames of pollen. The test colonies were allowed to fly freely, there were no nearby flowering crops and few flowering weeds (clover). Colonies were assembled according to treatment and groups were placed at least 20 m apart from each other. Two colonies (one control colony and one of the highest exposure rate colonies) (301 mg glyphosate a.e./L) became queenless after dosing but were retained in the study as the marked brood was viable and this was therefore not considered to have a significant impact on the study. All colonies were generally assessed within 1 week before dosing and again within weeks 1, 2, and 3 after dosing (day 0). Each assessment was carried out on every frame within each colony, and included counts of the number of combs of adults, brood (sealed and unsealed), and stores (nectar and pollen) as well as any behavioral or physical abnormalities.

The processes during the study followed the method for honeybee brood feeding test with insect growth regulating compounds (Oomen et al. 1992). Up to 24 h before dosing, 100 brood cells containing eggs, 100 cells containing 1- to 2-day-old larvae and 100 cells containing 3- to 4-day-old larvae were selected in each colony and marked using the standard Oomen et al. (1992) acetate overlay sheet method.

On day 0, one group was an untreated control, i.e., fed 1 L 50% sucrose solution, 3 groups were treated with glyphosate IPA salt (added to 1 L of 50% sucrose to achieve doses of 301, 150 and 75 mg glyphosate a.e./L), and one group was treated with the toxic reference, fenoxycarb, dispersed in 1 L of 50% w/v sucrose (750 mg a.s./L). Doses were administered by removing frames of stores from the colonies and placing a 1 L glass container containing the treated or control sucrose within the brood chamber. The container contained a cork float to allow access to the sucrose solution. Samples of each concentration of test item treated sucrose solution were retained for analysis by subsampling 5 mL from each of the prepared solutions and combining to a single sample (total 4 samples; control and 3 doses of glyphosate). The uptake of each sucrose solution was checked daily and the container removed when empty or after 5 days whichever was later.

On day 7, the marked brood cells (eggs, young, and old larvae) were assessed for mortality and appearance in each test colony. The final assessment for each larval was undertaken at day 13 for brood cells marked as containing old larvae, day 15 for cells containing young larvae, and day 16 for cells containing eggs. The cells were uncapped, the bee removed carefully with forceps, and the age of the bee assessed, weighed, and any deformities noted.

On days 4 and 7 (when the marked brood cells were assessed), samples of ten 4- to 5-day-old larvae were sampled from each treated colony (not from an area in which marked brood cells were located) for residue analysis. For the purpose of this study, mortality was defined as the total number of cells in any one group at any one observation period that were empty (other than recently emerged), contained dead larvae or pupae or contained larvae or pupae that were considered unhealthy (sick) and unlikely to survive. Brood mortality was statistically analyzed using a generalized linear model linked to a logit distribution for the brood mortality data and an analysis of variance for pupae weight data to determine the no observed effect concentration (NOEC) (equivalent to the no observed adverse effect level [NOAEL]) statistically, using the software Genstat v12 (VSN International). The study was considered valid if there were significant effects of the toxic reference (>40% effects on all stages) during the detailed brood assessment when compared to the control. The performance of the colonies in the control group were comparable with historical control data for the testing facility (10%–30% larval mortality overall), and demonstrate that the control colonies had performed correctly.

## RESULTS

### Exposure study

Daily assessments were made of the percentage of the plants that had wilted leaves or flowers. The crop started to show significant effects of the treatment from day 4 onward in both glasshouses and this coincided with the decreased foraging activity in glasshouse 2 although less pronounced effects on foraging were observed in glasshouse 1.

Foraging assessments showed foraging activity on the crop at the start of the study and this continued throughout the exposure period in glasshouse 1 with a peak on day 4; lowest foraging activity was on day 5 at 38% of the mean prespray activity. In glasshouse 2, the foraging activity declined throughout the assessment period and reached <10% of the mean prespray activity on days 5 to 7. The weights of pollen collected from the traps fitted to each hive ranged from 0.37 to 1.8 g per colony per day.

**Figure 1:**
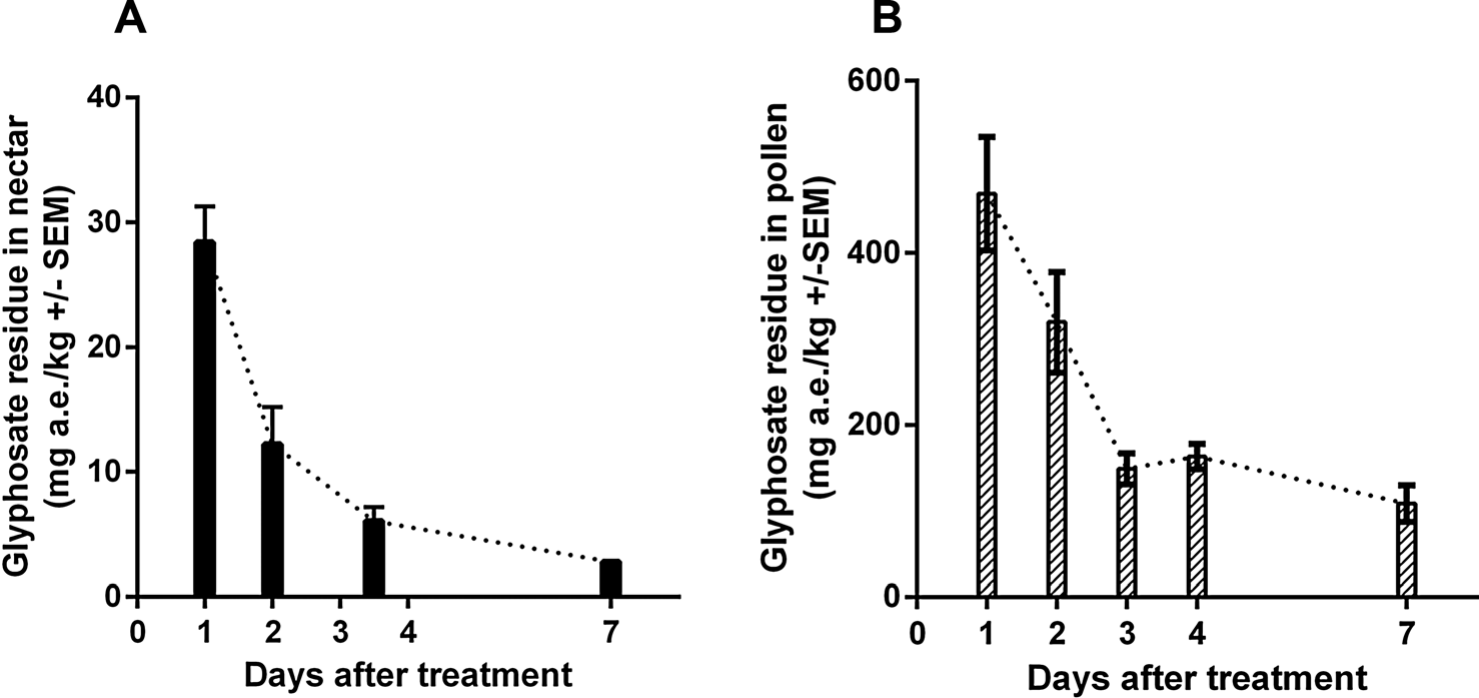
Decline of glyphosate residues (mg a.e./kg ± SE). (A) Nectar collected from foragers. The nectar sample from days 3 and 4 were combined due to the small amount collected for analysis. (B) Pollen collected in pollen traps in mg a.e./kg matrix.

Samples of honeybee products (nectar and pollen) and larvae were analyzed for residues of glyphosate acid equivalents. Glyphosate residues in nectar samples taken from forager bees before the application were not detectable (<0.3 mg a.e./kg). Residues in nectar samples taken at various time points after the application and originating from forager honeybees ranged from 2.78 to 31.3 mg a.e./kg and declined over time (Figure 1A). Residues in nectar samples taken from the colonies 7 days after the application ranged from below the LOQ (1.0 mg a.e./kg) to 1.30 mg a.e./kg (Table2).

**Table 2:** Exposure assessment of a brood study colony to glyphosate residues under 2 scenarios used to establish low- and mid-dose levels in bee brood study

Scenario	Daily intake of glyphosate residues in nectar (1944 g nectar/day) [mg]	Daily intake of glyphosate residues in pollen (9 g pollen/day) [mg]	Total daily intake of glyphosate residues [mg]	Uptake over 3 days [mg]	Adjustment from 2.88 kg a.e./ha to 2.16 kg a.e./ha [mg]^g^
Day 1 maximum mean residues (31.3 µg a.e./g in nectar; 573.5 µg a.e./g in pollen)	60.8^a^	5.2^b^	66.0	198	148.5^c^
Mean residues over days 1–3 (15.6 µg a.e./g in nectar; 310.1 µg a.e./g in pollen)	30.3^d^	2.8^e^	33.1	99.3	74.5^f^

The high dose for the study reflects twice the mid-dose level.

a Derived from 1.944 kg nectar consumed/d × 31.3 mg a.e./kg = 60.8 mg glyphosate a.e.

b Derived from 0.009 kg pollen consumed/d × 573.5 mg a.e./kg = 5.2 mg glyphosate a.e.

c Value of 148.5 mg was rounded to 150 mg to achieve the nominal mid-dose concentration in brood study.

d Derived from 1.944 kg nectar consumed/d × 15.6 mg a.e./kg = 30.3 mg glyphosate a.e.

e Derived from 0.009 kg pollen consumed/d × 310.1 mg a.e./kg = 2.8 mg glyphosate a.e.

f Value of 74.5 was rounded to 75 mg to achieve the nominal low-dose concentration in brood study.

g The determined residue content based on an application rate of 2.88 kg a.e./ha was adjusted to reflect the lower application to the rate of 2.16 kg a.e./ha.

Residues in pollen samples taken from the pollen trap before the application were not detectable (<0.3 mg a.e./kg). Residues in pollen samples taken at various time points after the application and originating from the trap ranged from 87.2 mg a.e./kg to 629 mg a.e./kg and declined over time (Figure 1B). Residues in larvae samples at 2 time points (day 4 and day 7) after the application ranged from 1.23 mg a.e./kg to 19.50 mg a.e./kg (Table2).

### Effects study

#### Consumption of treated sucrose

Analysis of the dosing solutions showed they were within 11% of the nominal doses. The control colonies consumed between 0.63 and 1.0 L of untreated sucrose. In the glyphosate-treated colonies, at least 3 of the 4 colonies in each group consumed the total volume of treated sucrose fed to each of them. There was no statistically significant difference in sucrose consumption in comparison to control for the 301 mg a.i./L group (*p* = 0.438), 150 mg a.i./L group (*p* = 0.212), the 75 mg a.i./L group (*p* = 0.054), which was slightly higher than the control, and the positive control fenoxycarb (*p* = 0.151).

In the 301 mg glyphosate a.e./L group, one colony consumed 0.39 L and the other 3 each consumed 1.0 L resulting in mean exposure to 255 ± 26 mg glyphosate a.e. In the 150 mg glyphosate a.e./L group, one colony consumed 0.67 L and the other 3 each consumed 1.0 L resulting in mean exposure to 130 ± 12 mg glyphosate a.e. In the 75 mg glyphosate a.e./L group one colony consumed 0.90 L and the other 3 each consumed 1.0 L resulting in mean exposure to 73 ± 2 mg glyphosate a.e. In the fenoxycarb treated colonies, consumption rates ranged from 0.45 to 0.88 L resulting in mean exposure to 510 ± 72 mg fenoxycarb. Exposure at the 150 mg a.i./L dose was significantly lower than at the 301 mg a.i./L dose (*p *= 0.049) and exposure at the 75 mg a.i./L dose was significantly lower than at 150 mg a.i./L dose (*p* = 0.002).

#### Brood mortality

**Figure 2:**
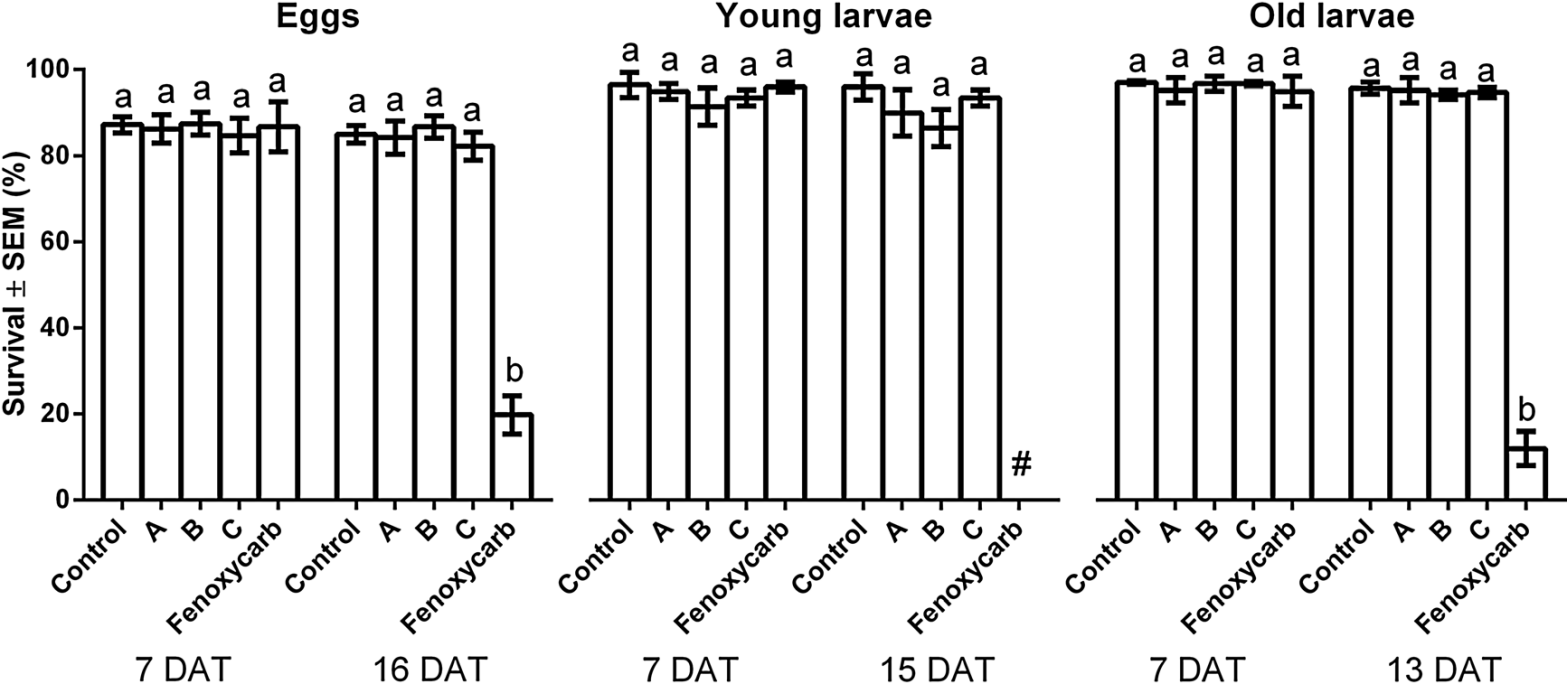
Survival (% ± SE) of Eggs (7 and 16 Days After Treatment, DAT), Young Larvae (7 and 15 DAT) and Old Larvae (7 and 13 DAT) for treatment groups (mean consumption) Control (0 mg glyphosate a.e.), A (255 ± 46 mg glyphosate a.e.), B (138 ± 12 mg a.e.), C (73 ± 2 mg glyphosate a.e.), and Fenoxycarb (510 ± 72 mg). Different letters above the bars indicate statistical difference (P < 0.05) from the respective control. ^#^ no statistical analysis as no variance due to 100% mortality.

Figure 2 summarizes the survival of marked brood stages at day 7 after dosing and just before emergence. There were no significant treatment-related effects except in the fenoxycarb toxic reference treated colonies, in which overall survival of marked cells was 20% for marked eggs (*p* < 0.001), 0% for marked young larvae (*p* < 0.001) and 12% for marked old larvae (*p* < 0.001), meeting the established validity criterion for the toxic reference (>40% effects at all stages). This can be compared with overall survival of 85% for marked eggs, 96% for marked young larvae, and 96% for marked old larvae in controls and 82%–87% for marked eggs (300 mg a.i./L: *p* *= *0.435, 150 mg a.i./L: *p* = 0.310, 75 mg a.i./L: *p* = 0.250), 87%–94% for marked young larvae (300 mg a.i./L: *p* = 0.185, 150 mg a.i./L: *p* = 0.060, 75 mg a.i./L: *p* = 0.254), and 94%–95% for marked old larvae (300 mg a.i./L: *p* = 0.434, 150 mg a.i./L: *p* = 0.202, 75 mg a.i./L: *p* = 0.291) in the glyphosate-treated colonies. The control mortality is similar to historical levels in studies conducted at the Food and Environmental Research Agency (FERA) (10%–30%). Deformities were observed in the fenoxycarb-treated colonies where discolored heads, thorax, and abdomens were noted. No deformities were observed in of the control or any glyphosate-treated colonies. Additionally, there were no significant effects on the mean weight of the exposed pupae (Table3) compared to controls in the 300 mg a.i./L group (*p* = 0.424), the 150 mg a.i./L (*p* *= *0.207), or the 75 mg a.i./L (*p* = 0.292). The fenoxycarb-treated colonies showed significant effects on weight of surviving pupae marked as old larvae (*p* = 0.003). The only dead pupae observed in any significant number were those in the fenoxycarb treated group where a mean of up to 190 pupae/day was observed and a mean of 600 pupae were recovered from the colonies over the 17-day period after dosing compared with 2.0 pupae/d in the control and 1.3 to 1.8 pupae/d in the glyphosate-treated colonies. The only adverse effects on colony development were observed in the fenoxycarb-treated colonies where declines in the numbers of bees and brood were observed in the latter stages of the study compared to controls for the 300 mg a.i./L group (*p* = 0.401), the 150 mg a.i./L group (*p* = 0.414), the 75 mg a.i./L group (*p* = 0.360), or the positive control fenoxycarb (*p* = 0.070).

**Table 3:** Mean pupae weight with SE at final assessment including dead and sick in the fenoxycarb treatment

Treatment	Dose rate mg/L	Mean dose consumed mg (SE)	Weight-surviving pupae marked as eggs (mg)	Weight-surviving pupae marked as young larvae (mg)	Weight-surviving pupae marked as old larvae (mg)
Control	0	0	127.5 ± 0.7	128.4 ± 0.6	128.9 ± 0.4
Glyphosate	301	255 ± 46	135.7 ± 0.6	125.4 ± 0.6	125.6 ± 0.4
Glyphosate	150	138 ± 12	126.7 ± 0.6	124.4 ± 0.8	122.6 ± 0.5
Glyphosate	75	73 ± 2	124.7 ± 0.8	128.3 ± 1.0	121.2 ± 0.5
Fenoxycarb	750	510 ± 72	125.9 ± 0.9	128.8 ± 1.3	115.4 ± 1.0^a^

SE = standard error.

a Statistically different effect (*p* < 0.01).

#### Adult bee mortality

No biologically significant adult mortality was observed in any treatment group with a mean total of 73 to 25 dead adult workers were recovered from dead bee traps over the entire 17-day period after dosing.

#### Residue analysis

**Figure 3:**
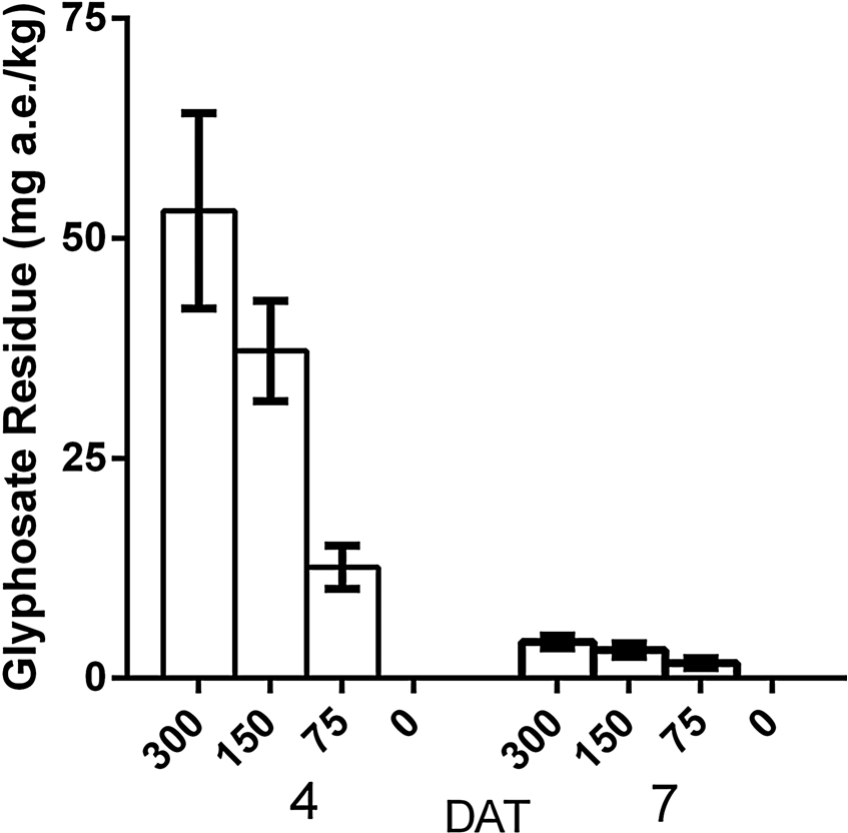
Residues (mg a.e./kg ± SE) in larvae 4 and 7 days after treatment (DAT) for dose groups with dose rate of 300, 150, 75, and 0 mg a.e./kg sucrose solution.

The residues in larvae sampled at 2 time points (day 4 and day 7) after dosing of the colonies (Figure 3) ranged from below the LOQ (1.0 mg a.e./kg) to 82.1 mg a.e./kg (at the highest dose rate) confirming that larvae were exposed to test item provided in the sucrose solution and consumed it. There was a linear relationship between dose level and glyphosate levels in larvae on days 4 and 7. Levels of day 7 were considerably lower than on day 4 and are likely the result of larval growth and glyphosate exposure ending after 5 days of exposure. Notably, these residue levels are comparable with values from the exposure study which ranged from 2.9 to 19.5 mg a.e./kg with a mean of 11.5 mg a.e./kg on day 4 to 1.2 to 10.6 mg a.e./kg with a mean of 5.3 mg a.e./kg on day 7 after the glyphosate application.

## DISCUSSION

There are few studies assessing the toxicity of herbicides to honeybee colonies (Moffett and Morton 1975; Ferguson 1988; Burgett and Fisher 1990) and very limited data quantifying exposure at the colony level (Thompson 2012). This study brought together a realistic worst-case exposure scenario in which residues of glyphosate were detected in samples of pollen and nectar collected from returning foragers and in samples of nectar and larvae collected from the hives with an assessment of the effects on honeybee brood exposed at these levels.

No adverse effects on adult bees or bee brood development were observed in any of the glyphosate-treated colonies. This confirms and extends the previous work on glyphosate that reported no effects on bee colonies fed up to 5 mg/kg glyphosate (Ferguson 1988) or when colonies were fed 1 L of 40% sucrose solution containing 5% Roundup® (a glyphosate-containing herbicide), mixed together with a spray drift retardant (Burgett and Fisher 1990), In the latter study, the concentrations fed to the treated bee hives were estimated 100 to 1000 times the environmentally relevant concentrations but there were no supporting residue data presented in either study. Similarly, no effects were reported after feeding picloram, 2,3,6 TBA or dicamba at 1000 mg/kg (Moffett and Morton 1975). However, complete cessation of brood rearing was observed when colonies were fed phenoxy herbicide formulations at 500 mg/kg, and a marked reduction in brood rearing occurred following exposure to 100 mg/kg (Moffett and Morton 1975). Previous studies have not related the dosages used in these studies to realistic worst-case exposure levels.

In this study, exposure rates of glyphosate were based on measured residues achieved in a glasshouse residue study. This is a worst-case realistic exposure in that the foraging bees were confined to the treated crop, and there was no spray drift during application or direct rainfall on the crop. However, the attraction of the crop declined after 4 days due to the action of the herbicide. There are few studies that have assessed exposure of honeybee colonies to herbicides. Peak glyphosate residues after application at 2.88 kg a.e./ha were 31.3 mg a.e./kg nectar and 574 mg a.e./kg pollen and mean residues over the first 3 days of exposure were 15.6 mg a.e./kg nectar and 310 mg a.e./kg pollen. Mullins et al. (2010) reported levels of 15 different herbicides in pollen and bees sampled from North American honeybee colonies with highest residues (pendimethalin) 1.7 mg/kg pollen. However, these data do not represent targeted monitoring that may result for close temporal–spatial application of pesticides. Also, there are no robust monitoring data available for glyphosate in bee-relevant plant matrices, because the focus for this kind of monitoring is usually rather on insecticides than on herbicides. The most recent data from the United States suggest herbicide residues in honeybee-collected pollen ranged from a mean of 0.16 (max 1.6) μg carfentrazone ethyl/kg to a mean of 5.16 (max 69.5) μg pendimethalin/kg (Pettis et al. 2013). The residue per unit dose (RUD) approach for sprayed pesticides predicts maximum residues of 59.6 mg a.e./kg (RUD in nectar from EFSA, 2013 × application rate, or 20.7 × 2.88) mg a.e./kg nectar and 431 (RUD in pollen from EFSA, 2013 × application rate, or 149.8 × 2.88) mg a.e./kg pollen (EFSA 2013). The US Environmental Protection Agency (USEPA 2012) approach uses an RUD for tall grass to predict maximum residues in nectar and pollen resulting in a predicted maximum of 282 mg/kg nectar and 282 mg/kg pollen, whereas the data in this study and those reviewed by EFSA (2013) suggest far lower residues are observed in nectar than pollen. The residues in larvae 4 days after the start of dosing (12.7 ± 2.5 mg a.e./kg larva) at the 3-day mean residue levels (based on 2.16 kg a.e./ha) were similar to those identified in the exposure study where no other forage was available (11.9 ± 3.8 mg a.e./kg larva with an application rate of 2.88 kg a.e./ha). This suggests that the proposed equations to predict exposure of larvae that are based on residue and consumption data for larvae and brood levels per colony were robust. Peak residues in larvae sampled at 2 time points (day 4 and day 7) after the introduction of sucrose solution treated at the 3-day mean residues to the hives were 18.4 mg a.e./kg larva. These can be compared with those published by EFSA (2013) of 9.5 (4.4 × 2.16) μg a.e./larvae—based on a larvae weighing 0.1 g, this is equivalent to 95 mg a.e./kg larvae over the 5-day developmental period or 19 mg a.e./kg larva/day based on complete metabolism and/or irreversible binding of ingested glyphosate within a 24 h period.

Previous studies have used the Oomen et al. (1992) methodology primarily to identify insect growth-disrupting properties of pesticide formulations. Studies specifically designed to identify mode of action are less reliant on identifying realistic exposure scenarios (de Ruijter and van der Steen 1987). This study has developed methodology that can be used to identify the appropriate dose rates for use in colony feeding studies based on realistic worst-case residues in pollen and nectar. Such an approach can be used to determine dose–response effects of colony-level exposure so as to limit the number of replicated tunnel or field-scale studies that need to be undertaken and may be of particular benefit where residues in pollen and nectar are crop- and/or formulation-specific such as systemic seed treatments and granular applications.
